# Development and validation of hypermethylated gene markers in cervical cytological samples for detecting endometrial cancer (EndoMethy-I trial)

**DOI:** 10.3389/fonc.2026.1849730

**Published:** 2026-06-19

**Authors:** Xiaojing Chen, Lixin Liu, Xitong Jin, Yanan Cheng, Huanwen Wu, Yan You, Yuligh Liou, Pei Liu, Jinghe Lang, Lei Li

**Affiliations:** 1 Department of Obstetrics and Gynecology, Peking Union Medical College Hospital, Beijing, China; 2 National Clinical Research Center for Obstetric and Gynecologic Diseases, Beijing, China; 3 State Key Laboratory for Complex, Severe and Rare Diseases, Peking Union Medical College Hospital, Beijing, China; 4 Department of Pathology, Peking Union Medical College Hospital, Beijing, China; 5 Department of Medical Laboratory, Beijing Origin-Poly Bio-Tec Co., Ltd., Beijing, China; 6 Department of Obstetrics and Gynecology, Beijing Tsinghua Changgung Hospital, Beijing, China

**Keywords:** *CDO1*, *CELF4*, cervical cytology, DNA methylation, endometrial cancer, transvaginal ultrasound

## Abstract

**Objectives:**

To explore hypermethylated gene markers in cervical scraped cells that may be associated with endometrial cancer and validate their diagnostic role in endometrial cancer.

**Methods:**

In an exploratory cohort consisting of 40 paired endometrial tissue and cervical scraping samples, high-performance methylation-targeted genes associated with endometrial cancer were identified. In training and validation sets with 347 and 149 participants, respectively, methylated markers from cervical cytology together with other epidemiological and clinical parameters were assessed to determine their accuracy in detecting endometrial cancer. A decision tree was constructed using methylation markers, bleeding symptoms and endometrial thickness on transvaginal ultrasound (TVS).

**Results:**

In this exploratory study, eleven genes highly related to endometrial cancer were evaluated using a methylation array. In the training cohort, the highest AUC values for detecting endometrial cancer were 0.93, 0.91, and 0.89 for hypermethylated *CDO1*, *NEFM*, and *CELF4*, respectively. In the validation set, combining methylated *CDO1*, *CELF4*, and *NEFM* achieved a sensitivity of 94.6% (95% confidence interval 85.1-98.9) and a specificity of 92.8% (89.3-95.5) for detecting endometrial cancer. Integration of the endometrial thickness of TVS slightly improved the diagnostic specificity with very low sensitivity.

**Conclusions:**

Cervical cytological DNA methylation assays of *CDO1*, *CELF4* and *NEFM* provide a reliable and safe strategy for detecting endometrial cancer and are superior to other noninvasive evaluation methods or parameters.

## Introduction

Endometrial cancer, the most common gynecological cancer, is increasing in incidence worldwide, especially in high-income countries and cities (1). Increased risk of endometrial cancer is associated with a history of unopposed estrogen therapy, obesity, diabetes, and Lynch syndrome (2–5). Endometrial cancer is predominantly identified in women who present with abnormal uterine bleeding (AUB) or postmenopausal bleeding. Nonetheless, after invasive procedures and evaluations, only 0.33% of AUB patients and 9% of postmenopausal women are diagnosed with endometrial cancer (6, 7). Endometrial cancer severely impairs fertility, and an increasing incidence of the disease and its precursor lesions has been discovered among younger women (8).

Currently, the use of noninvasive screening or diagnostic methods for endometrial cancer remains limited in clinical practice, despite promising developments in molecular approaches (9). Clinical evaluation involves the use of transvaginal ultrasound (TVS) in conjunction with endometrial biopsy (10–12). TVS is inexpensive and convenient and provides invaluable insight into endometrial conditions such as thickening of the endometrium, the presence of intrauterine masses or lesions, and potential signs of distal metastasis. TVS, as well as other imaging methods, had limited sensitivity and specificity, highlighting the critical role of endometrial biopsy for accurate diagnosis. Biopsy approaches, including dilatation and curettage, hysteroscopy and other invasive interventions, are inconvenient and costly and are associated with potential complications (13, 14). It is unfeasible to recommend invasive histological evaluation as a first-line evaluation method. No guidelines recommend biomarkers as effective screening methods (15).

Recently, multiomics has provided promising proposals and translational methods for screening for endometrial cancer. These plans consisted of mutation detection (16), mRNA modification (17), metabolomics (18), and epigenetics. Methylation assays have made essential progress and have become the most promising screening tool because of their great accuracy, reliability and low cost. DNA methylation, an epigenetic modification that regulates gene expression, plays a key role in normal development, gene silencing, and genomic imprinting (19). Aberrant hypermethylation of gene promoters is a common mechanism for tumor suppressor gene inactivation, and widespread alterations in DNA methylation are hallmarks of malignant transformation (20–22). Importantly, many of these epigenetic changes occur early in tumorigenesis, making them attractive candidates for early cancer detection (22, 23). In the context of endometrial cancer, multiple hypermethylated genes, including *CDO1*, *CELF4*, and *NEFM*, have been identified as potential molecular biomarkers (24, 25). Moreover, DNA methylation analysis is applicable to a wide range of sample types, including blood, urine, feces, and cervical cytology, enabling minimally invasive or noninvasive screening approaches (26). Methylation assays for detecting endometrial cancer in cervical cytological samples have been successful in several studies.

In this study, we developed methylation markers for targeted genes, and on the basis of an assessment of cervical cytology, we validated their efficacy in the detection of endometrial cancer with or without clinical parameters.

## Methods

### Ethical approval

The Institutional Review Board of the study center approved the study (No. ZS-2740). All patients provided their consent before enrollment. The Cell-free DNA Methylation for Endometrial Cancer (EndoMethy-I) study registration number is NCT04651738 (*clinicaltrials.gov*, registered on November 26, 2020). All procedures performed in the study involving human participants were in accordance with the ethical standards of the institutional and National Research Committee and with the 1964 *Declaration of Helsinki* and its later amendments or comparable ethical standards.

### Study design and patient enrollment

**Figure 1:**
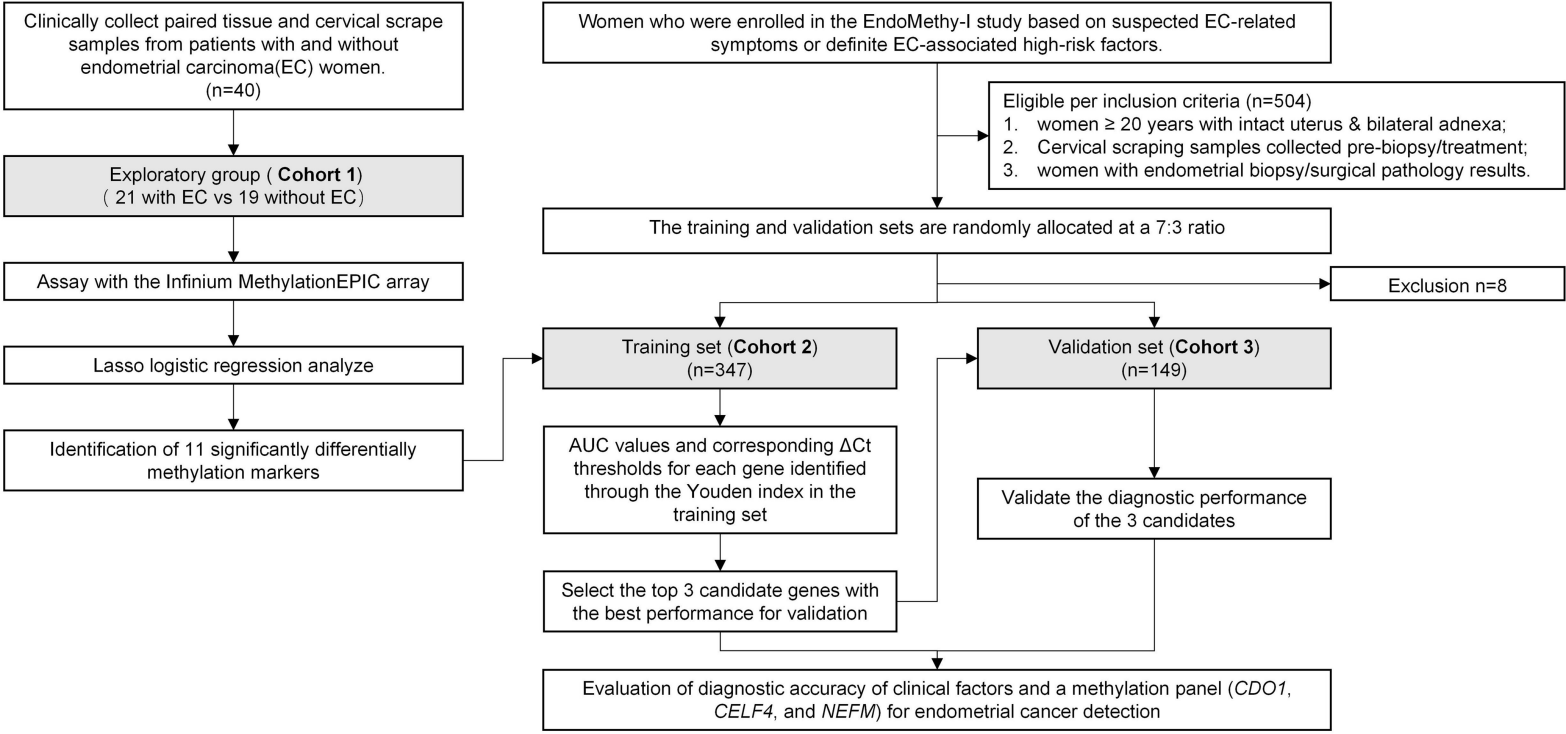
Flow chart of the study enrollment and participants.

The study was a prospective observational cohort study conducted at a single center and included three cohorts (**Figure 1**). Cohort 1 was an exploratory cohort consisting of 40 participants with and without endometrial cancer. Their endometrial tissues and cervical scraping cytology results were collected and analyzed to identify genes targeted as methylation markers. Other participants were enrolled based on the presence of suspected endometrial cancer-related symptoms or definite endometrial cancer-associated high-risk factors; these participants were randomly divided into a training set (cohort 2) and a validation set (cohort 3) at a ratio of 7:3. The methylation markers present in these two cohorts were further tested via cervical cytology to determine the accuracy of detection of endometrial cancer.

The inclusion criteria were as follows: more than 18 years of age, possessing an intact uterus, presenting with suspicious symptoms (such as AUB or postmenopausal bleeding) or other risk factors for endometrial cancer, and having available endometrial histology. Women were excluded from the study if they were pregnant, had a history of hysterectomy, had malignancies or their precursors of low genital tracks, or had active cancer under treatment.

### Clinical and histological evaluations

Epidemiological and clinical data, including body mass index (BMI), history of diabetes and polycystic ovary syndrome, and biomarkers (CA125) if available, were collected from medical reports and/or interviews with the participants. All participants reported bleeding symptoms, including abnormal uterine bleeding (AUB) or postmenopausal bleeding. Within the month prior to histological evaluation, all participants underwent TVS for assessment of endometrial thickness.

Endometrial histology was performed by endometrial biopsy, hysteroscopy, or hysterectomy. The histological results were independently reviewed as standards to develop and validate methylation markers.

### Sample collection

Before any examination procedures were performed, cervical scraping cell specimens were collected using a Cervex-Brush^®^ (Rovers Medical Devices) and stored in ThinPrep^®^ liquid-based thin layer cell preservation solution (Hologic, Marlborough, MA, USA) at room temperature. These cell samples were concentrated, and the cells were immediately placed in RNAlater Stabilization Solution (Ambion, Thermo Fisher Scientific) before being stored at -80 °C until analysis.

In the exploratory set, endometrial tissues (malignancies, benign lesions or normal endometrium) were collected during the participants’ surgical procedures and preserved in a freezer at -80 °C.

### Development of the methylation assay

Endometrial tissue was ground with a mortar and pestle in liquid nitrogen and subsequently added to 500 μl of 0.1 M phosphate-buffered saline to form a cell suspension. Genomic DNA was extracted from tissue cell suspensions or scraped cervical cells using a TIANamp Genomic DNA Kit (Tiangen Co., Beijing, China) according to the manufacturer’s protocol. A NanoDrop 2000c spectrophotometer (Thermo Fisher Scientific, MA, USA) was used to quantify the amount of extracted DNA. Afterward, 500 ng of genomic DNA was subjected to bisulfite conversion using EZ DNA Methylation-Gold Kits (Zymo Research, CA, USA) according to the manufacturer’s recommendations.

In the exploratory set, methylation data from paired scraping cells and tissue samples were processed using the Infinium MethylationEPIC array (Illumina, San Diego, CA, USA) (27). The raw data were processed using the minfi R package (v1.44.0). Quality control was performed by checking sample-level median methylation intensity and CpG-level detection p-values (threshold: p < 0.01). Samples in which >1% of CpGs failed to be detected were excluded. Normalization was performed using the Noob (normal-exponential out-of-band) background correction method followed by BMIQ (beta-mixture quantile normalization) to correct for probe-type bias. CpGs located on sex chromosomes and known SNP-associated probes were removed. Beta values (ranging from 0 to 1) representing the proportion of methylated molecules were used in the downstream analysis. We applied Lasso logistic regression to analyze the methylation data, enabling robust feature selection and identification of key methylation markers associated with differentiation between the case and control groups. To ensure the reliability of our findings, we incorporated 10-fold cross-validation during the modeling process, which facilitated a thorough evaluation of model performance and prevented overfitting. We then validated the differentially methylated positions (DMPs) identified through Lasso logistic regression, along with the CpGs located 500 bp up- or downstream, to confirm their significance as markers of differential methylation between cases and controls. On the basis of this analysis, we designed PCR primers for reactions targeting the regions where the index CpG and surrounding CpGs exhibited the most significant differences, and at least one CpG had no or very low methylation in the control. In addition, only CpG sites showing hypermethylation in endometrial cancer cases relative to controls were considered, consistent with the study’s focus on cancer-associated promoter hypermethylation. Sites exhibiting the opposite pattern were excluded from the analysis regardless of the magnitude of the difference in methylation.

### Target region and CpG island identification for primer design

Prior to EPIC array analysis, eleven candidate genes were preselected through a structured literature review based on the following predefined criteria (1): prior published evidence of aberrant promoter hypermethylation in endometrial cancer or other gynecological malignancies (2); reported functional association with tumor suppressor activity or cancer-related gene silencing; and (3) the presence of CpG-rich promoter regions suitable for bisulfite-based methylation-specific PCR analysis (the genes CDO1, NEFM, CELF4, ACLY, G3BP2, LOC100133985, MAPK9, PPP1R3G, AP1G2, TSPYL1 and MX1). Among these genes, CpG-rich regions were identified, and primer sets were designed using Primer Premier 5.0 (Premier Biosoft Ltd., Palo Alto, USA). The designed primer sets included a forward primer (F), a reverse primer (R), and a TaqMan probe (FR), which were designed under stringent design conditions. The primers were designed to be within the length range of 20–25 nucleotides, ensuring specificity, while the melting temperature was targeted between 58 °C and 62 °C to provide uniform annealing conditions. To maintain a balance between stability and specificity, the GC content was optimized between 45% and 55%. During the design process, additional checks were conducted to avoid secondary structures such as primer–dimer formations and hairpin loops.

Given that bisulfite conversion alters DNA sequences, the primers and the TaqMan probe were specifically optimized for bisulfite-converted, methylated DNA. This optimization included labeling the TaqMan probe with a fluorescent reporter dye at the 5’ end and a quencher dye at the 3’ end to ensure that the fluorescent signal accurately represented the methylation status of the genetic targets.

### Quantitative methylation-specific PCR

Within the training set, a subset was designated as the inner training set, which was used to confirm the optimal number of CpG sites for subsequent analysis. Independent methylation QPCR was performed for 11 candidate genes. We prepared a 25 µl reaction mixture containing 5 µl bisulfite-converted DNA, 5 µl primer and probe mixture, and 15 µl Master Mix. PCR products were amplified using the SLAN Real-Time PCR System (Hongshi Medi Tech Co., Shanghai, China) under the following cycling conditions: 96 °C for 10 min; 45 cycles of 94 °C for 15 s, 64 °C for 5 s, and 60 °C for 30 s; and a final extension at 25 °C for 1 min. Two crossing point (Cp) values were obtained, one from the target gene and another from GAPDH. The DNA methylation level was subsequently determined from the difference between the 2 Cp values (ΔCp = Cp_target_ gene − Cp _GAPDH_).

### Statistical analysis

All the statistical analyses were conducted using R version 4.2.1 (2022–06–23). Receiver operating characteristic curves, areas under the curve, and corresponding 95% confidence intervals were generated using the pROC package, version 1.18.0. Sensitivity, specificity, positive predictive value (PPV), and negative predictive value (NPV), along with their 95% confidence intervals, were calculated using the epi.tests and BDtest functions in the epiR (version 2.0.38) and bdpv (version 1.3) packages, respectively. Categorical variables are presented as numbers with percentages, and continuous variables are presented as medians with interquartile ranges (Q1–Q3) or means with standard deviations. Categorical variables were analyzed using the chi-square test or Fisher’s exact test, as appropriate. For comparisons of continuous variables between two independent groups, either an unpaired Student’s t test (for normally distributed variables) or the Mann–Whitney U test (for nonnormally distributed variables) was applied. When paired data were compared, either the paired Student’s t test (for normally distributed variables) or the Mann–Whitney U test (for nonnormally distributed variables) was used. Two-tailed *p* values <0.05 were considered to indicate statistical significance.

## Results

### Study flow and participants’ characteristics

**Figure 2:**
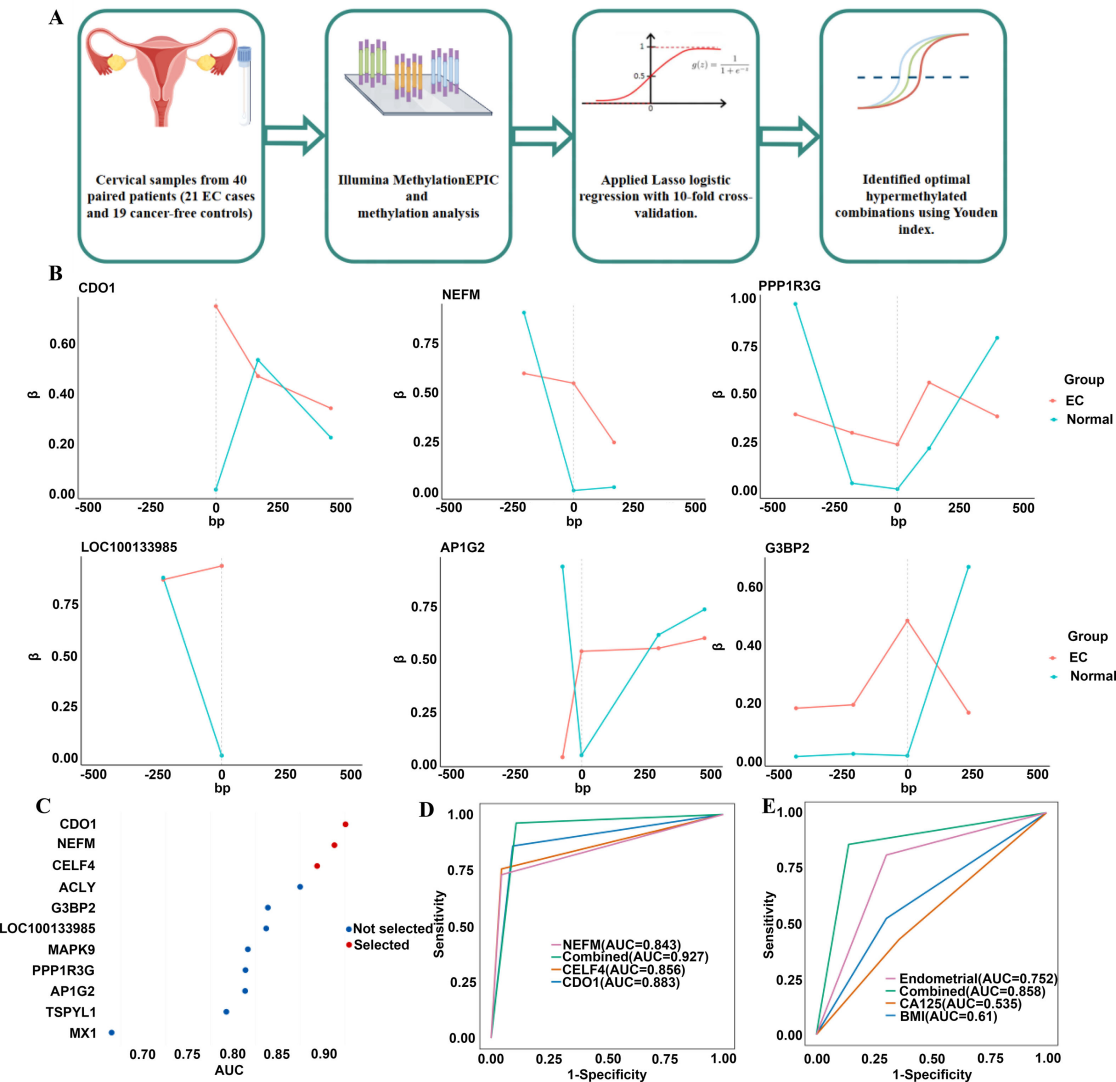
Overview of the study and selection of target genes. **(A)** Schematic workflow of marker development. The test was developed by epigenome-wide analysis of cervicovaginal samples from cancer cases and controls, and thresholds were fixed in a training set. **(B)** Example plots of selected CpG beta (methylation) values in control samples and endometrial cancer cases versus nearby CpGs (within ±500 bp). **(C)** Area under the curve (AUC) values of individual reactions for the discrimination of controls and patients with cancer in the training set. **(D)** Receiver operating characteristic (ROC) curves of different methylation combinations in the validation set. **(E)** ROC curves of different epidemiological and clinical parameters in the whole testing set.

The study flow is illustrated in **Figure 1**. There were 40 patients with paired endometrial issues and cervical scraping cytology, 21 and 19 patients with endometrial malignancies and benign endometrial findings, respectively. A diagram of the process and validation of the DNA methylation data is shown in **Figure 2**. The clinical characteristics of methylation chip testing in the exploratory cohort are listed in **Supplementary Table 1**.

As shown in **Table 1** and **Figure 1**, 496 patients with definitive endometrial histological outcomes were allocated to the training set (n=347) or validation set (n=149), with eight patients excluded. There were no significant differences between the training and validation sets in any of the analyzed variables, including age, histopathological classification, gravidity, parity, BMI, bleeding complaints, endometrial thickness, diabetes, PCOS, CA125 levels, or methylation status.

**Table 1:** Epidemiological and clinical characteristics of participants in the training and validation sets.

Characteristics	Training set n (%)	Validation set n (%)	*p* value
Participants (n)	347	149	
Age (years, median [SD])	47.49 (11.46)	46.94 (10.99)	0.617
Uterine histology			0.169
Normal	252 (72.6)	122 (81.9)	
EIN	9 (2.6)	3 (2.0)	
EC	78 (22.5)	21 (14.1)	
Other cancer	8 (2.3)	3 (2.0)	
Gravidity			0.555
0	45 (13.0)	23 (15.4)	
≥1	302 (87.0)	126 (84.6)	
Parity			1.000
0	302 (87.0)	126 (84.6)	
≥1	81 (23.3)	35 (23.5)	
BMI at diagnosis (kg/m^2^)			0.680
<25	219 (63.1)	100 (67.1)	
25–29.9	100 (28.8)	39 (26.2)	
≥30	28 (8.1)	10 (6.7)	
Bleeding symptoms			0.966
Post-menopause			
PMB	53 (15.3)	21 (14.1)	
No symptoms	48 (13.8)	22 (14.8)	
Pre-menopause			
Abnormal bleeding	144 (41.5)	64 (43.0)	
No symptoms	102 (29.4)	42 (28.2)	
Endometrial thickness			0.888
Post-menopause			
≥5 mm	50 (14.4)	23 (15.4)	
<5 mm	51 (14.7)	20 (13.4)	
Pre-menopause	119 (32.0)	50 (40.3)	
≥11 mm	68 (19.6)	33 (22.1)	
<11 mm	178 (51.3)	73 (49.0)	
Diabetes			1.000
Yes	20 (5.8)	8 (5.4)	
No	327 (94.2)	141 (94.6)	
PCOS			0.190
Yes	128 (36.9)	65 (43.6)	
No	219 (63.1)	84 (56.4)	
CA125 (U/ml)			1.000
<35	235 (75.3)	100 (75.8)	
≥35	77 (24.7)	32 (24.2)	
Methylation assessment			
CDO1m			0.162
Positive	92 (26.5)	30 (20.1)	
Negative	255 (73.5)	119 (79.9)	
CELF4m			0.236
Positive	71 (20.5)	23 (15.4)	
Negative	276 (79.5)	126 (84.6)	
NEFMm			0.159
Positive	69 (19.9)	21 (14.1)	
Negative	278 (80.1)	128 (85.9)	

BMI, body mass index; CA125, serum carbohydrate antigen 125; CDO1m, methylation assessment of *CDO1* gene; CELF4m, methylation assessment of *CELF4* gene; EC, endometrial cancer; EIN, endometrial intraepithelial neoplasia; NEFMm, methylation assessment of NEFM gene; PCOS, polycystic ovary syndrome; PMB, post-menopausal bleeding.

### Selection of hypermethylation markers in candidate genes

After adjustment of the false discovery rate in the Lasso logistic regression, we identified 12 CpG sites that were significantly differentially methylated between patients with endometrial cancer (endometrial cancer) and controls. One CpG site (cg01855118) was excluded from further analysis because of the inability to annotate it to a gene (**Supplementary Table 2**). Additionally, we examined other CpG sites located within ±500 bp of the identified DMPs, and the results are illustrated in **Figure 2B**. Upon identifying the differentially methylated CpG sites, we proceeded to design PCR assays that included primer sets and a standard buffer solution for the 11 selected sites and incorporated them into the training set (**Supplementary Table 3**). In the training set, the AUC and cutoff values of the eleven hypermethylated genes determined using the Youden index were highest for the CpG sites in *CDO1*, *NEFM*, and *CELF4*, at 0.93, 0.91, and 0.89, respectively (**Figure 2C**, **Supplementary Table 4**). Consequently, *CDO1*, *NEFM*, and *CELF4* were included in the training set.

### Diagnostic performance of DNA methylation in the training set

The characteristics of the participants in the training set are presented in **Supplementary Table 5**. In the training set, we assessed the diagnostic performance of various clinical and methylation indices for detecting endometrial cancer, as summarized in **Supplementary Table 6**. The individual AUC values for *CDO1^m^*(+), *CELF4^m^*(+), and *NEFM^m^*(+) were 88.30%, 85.59%, and 84.31%, respectively. When these three biomarkers were combined, the diagnostic accuracy improved further, achieving an AUC of 92.69% (**Figure 2D**). The positive results for any single gene or combination of methylation markers were significantly greater than those for BMI, CA125 level, or endometrial thickness. Additionally, the combination of these three biomarkers demonstrated high sensitivity and specificity. For instance, when at least one gene was positive, the sensitivity reached 96.15%, with a specificity of 89.22%. In contrast, the sensitivity of the single biomarker CA125 was only 53.85%, with a specificity of 69.89%, whereas the sensitivity for endometrial thickness was 55.13%, and the specificity was 72.12%.

### Diagnostic performance of DNA methylation in the validation set

The participant characteristics in the validation set are presented in **Supplementary Table 7**. In the validation cohort (n=149, including 21 endometrial cancer cases and 128 controls), significant intergroup differences were found for age, BMI, presenting complaint, endometrial thickness, diabetes mellitus, and serum CA125 level. As shown in **Supplementary Table 8** and **Figures 2E**, single-methylation gene analysis of *CDO1*, *CELF4* and *NEFM* or their combinations yielded greater diagnostic accuracy than did CA125 level, BMI or endometrial thickness in TVS. Specifically, NEFM showed the highest single-gene performance, with an AUC of 86.14%, outperforming both CDO1 (AUC 79.85%) and CELF4 (AUC 79.82%). The combination of CDO1 and NEFM achieved the best overall diagnostic accuracy (AUC 86.61%), with a sensitivity of 85.71% and a specificity of 87.5%, while the triple combination of CDO1, CELF4 and NEFM maintained a high AUC of 85.83%. Notably, all three methylation markers individually achieved significantly higher specificities (≥88.28%) than did CA125 level (64.06%) and BMI (≤69.53%), making them more reliable for ruling out false-positive results.

### Decision tree analysis for endometrial cancer

**Figure 3:**
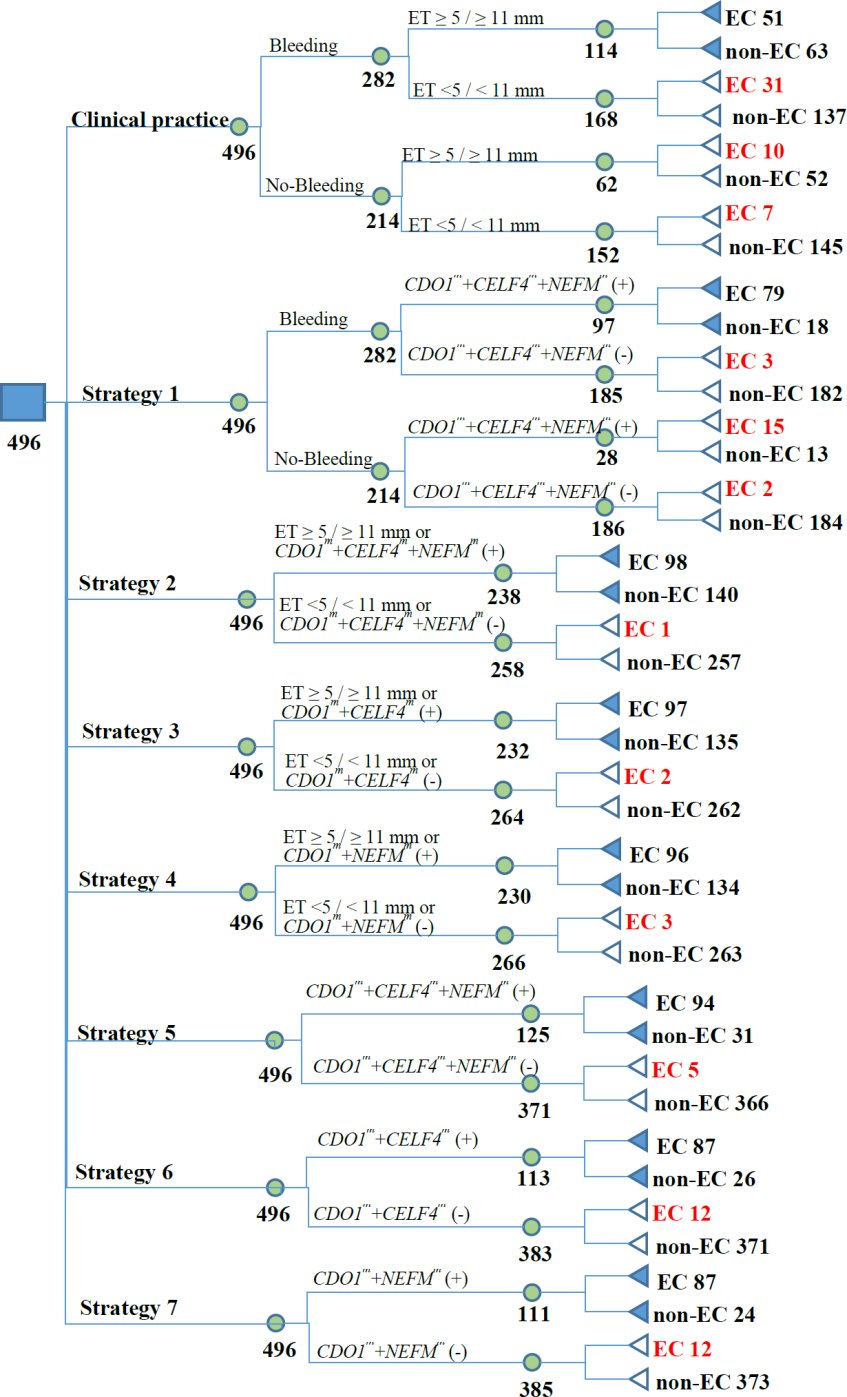
Decision tree and analysis of clinical practice and strategies for the diagnosis of endometrial cancer. One clinical practice strategy, four cotesting strategies (methylation combined with TVS), and three methylation strategies were compared using a decision tree. A positive result for methylated *CDO1* (*CDO1^m^*) is defined as a ΔCp ≤8.4. A positive result for methylated *CELF4* (*CELF4^m^*) is defined as a ΔCp ≤8.8. A positive result for methylated *NEFM* (*NEFM^m^*) is defined as a ΔCp ≤8.8. ET ≥5 was defined as an endometrial thickness ≥5 mm in postmenopausal women with positive results. An ET ≥11 mm was defined as an endometrial thickness ≥11 mm for premenopausal women with positive results.

As shown in **Figure 3**, a decision tree was constructed with methylated *CDO1*, *CELF4* and *NEFM* and bleeding symptoms and endometrial thickness on TVS. Definitions of diagnostic strategies and numbers of missed cancer cases are listed in **Supplementary Table 9**. The cotest Strategies 2, 3 and 4, i.e., combined methylation genes and TVS, had the least missed cancer cases, although with approximately 50% hysteroscopy rates. In Strategies 2 and 3, the two missed cases were young women (31.8 and 32.2 years of age); both had Lynch syndrome with immunohistochemical results of MLH-1 (+), MSH-2 (+), MSH-6 (+), and PMS-2 (+). In all participants, screening with only methylation markers resulted in five cases of missed cancer and a low hysteroscopy rate of 25.0%. A screening flow for endometrial cancer without methylation analysis (Current clinical practice) or a flow-only screening for women with abnormal bleeding (Strategy 1) is not acceptable because of their high missed diagnosis rates.

## Discussion

This study identified new hypermethylated genes in endometrial cancer using cervical scraped cells, aimed at differentiating malignant from nonmalignant cases, and evaluated their clinical performance during validation. Three methylation tests (*CDO1^m^*, *CELF4^m^*, and *NEFM^m^*) demonstrated high sensitivity and specificity, with values of 96.2% and 89.2%, respectively. Similarly, the sensitivity and specificity of the dual methylation test (*CDO1^m^* + *CELF4^m^*) were 92.34% and 89.2%, respectively. These results suggest that noninvasive combined methylation tests are reasonable diagnostic options for women with suspected endometrial cancer.

Women experiencing AUB, especially those with postmenopausal bleeding (PMB), may have a significantly increased association with endometrial cancer (28, 29). However, despite the increased risk, only 9% of women with PMB were diagnosed with endometrial cancer. However, only 9% of women with PMB are biopsy-diagnosed with endometrial cancer (7, 29). The American College of Obstetricians and Gynecologists (ACOG) Committee highlights vaginal bleeding as a primary symptom of endometrial cancer and encourages individuals to seek medical attention if their symptoms worsen. They also recommend TVS as sufficient for the initial evaluation of postmenopausal bleeding (10). TVS, a widely used first-line noninvasive diagnostic method for assessing endometrial thickness and identifying potential abnormalities within the uterus, serves as the cornerstone of initial diagnostic evaluations for the early detection and management of endometrial cancer in hospitals. The sensitivity of TVS exceeds 90% in detecting endometrial cancer (endometrial cancer) when endometrial thickness cutoffs are set between 3 and 5 mm, demonstrating its effectiveness as a first-line screening modality, as recommended by current guidelines (10, 30). Unfortunately, differences in pathologists’ experience make it challenging to maintain consistent results. Repeated invasive examinations may not only yield unclear results but also cause pain, inconvenience, and increased costs. A 5-mm cutoff value for endometrial thickness demonstrated lower sensitivity, measured at 77.1% (67.8–84.3%), and a specificity of 85.8% (85.7–85.9%) in postmenopausal women. Conversely, an 11-mm cutoff value in premenopausal women resulted in a sensitivity and specificity ranging from 60% to 92% and from 62% to 93%, respectively (31, 32). In contrast, the dual methylation test (CDO1m + CELF4m) in our study achieved a sensitivity of 92.34% and a specificity of 89.2%, markedly outperforming detection based on endometrial thickness. These findings suggest that use of the dual methylation assay could facilitate early detection and clinical management by nurses and clinicians in community health settings.

Biopsy pathology from endometrial curettage or hysteroscopy remains the standard process in the diagnosis of endometrial cancer, but it has limitations such as sampling invasiveness, sampling errors, poor repeatability, complications, and pathological experience (33, 34). In the validation set of this study, compared with other tests, such as CA125 level, BMI and TVS, either single or combined methylation genes of *CDO1*, *CELF4*, and *NEFM* demonstrated high sensitivity (66.67–85.71%) and high specificity (85.94–96.09%). These findings underscore the potential of the methylation test, suggesting that it could be considered a noninvasive option for detecting endometrial cancer. From a clinical implementation perspective, the methylation-specific qPCR assay described in this study features a streamlined three-step workflow: extraction of DNA from liquid-based cytology samples, bisulfite conversion, and real-time PCR amplification using TaqMan probes. Each of these steps can be performed using commercially available kits and standard molecular laboratory equipment without a need for specialized platforms or advanced bioinformatics infrastructure. The estimated turnaround time from sample receipt to reportable result is approximately 4–6 hours; thus, the method is compatible with routine outpatient diagnostic timelines. Importantly, the cervical scraping samples are collected using a Cervex-Brush during a standard gynecological examination (at the same visit at which liquid-based cytology or HPV testing is performed), thereby introducing no additional invasive procedure or clinical appointment for the patient.

Numerous studies suggest that DNA methylation, a reversible epigenetic modification in contrast to the permanent nature of genetic changes, serves as a valuable tool for diagnosis, monitoring, and determining treatment options, thereby aiding in clinical decision-making for cancer (35, 36). A meta-analysis on the accuracy of DNA methylation detection in endometrial cancer screening, which included 31 genes, such as ADCYAP1, ASCL2, BHLHE, HAND2, *CDO1*, and *CELF4*, identified these genes as reasonable biomarkers for endometrial cancer detection, with AUC values ranging from 0.80 to 0.97 (37). Chiara Herzog et al. reported that other GYPC and ZSCAN12 genes can be used to detect endometrial cancer with high sensitivity (97.2% to 100%) and specificity (75.8% to 89.1%) in cervical, self-collected, and vaginal swab samples from symptomatic patients (38). The accuracy of *CDO1* and *CELF4* methylation in cervical cytology samples for endometrial cancer is being assessed in trials conducted in China (36), which include endometrial cancer-METHY1-4, to obtain a comprehensive understanding of the effectiveness of methylation markers in cancer screening. Some findings from individual studies focusing on menopausal women with abnormal uterine bleeding, women with postmenopausal bleeding, and premenopausal women with abnormal uterine bleeding have been reported (24, 38–40). Here, the *CDO1*, *CELF4*, and *NEFM* methylation genes within endometrial cancer-Methy-1 were identified for the first time. The high specificity of *CDO1^m^* and *CELF4^m^* alone or in combination makes them ideal triage tools for detecting suspected endometrial cancer in a clinical gynecological setting. Moreover, the triple methylation test (*CDO1^m^*, *CELF4^m^*, and *NEFM^m^*) exhibits high sensitivity (96.2%), specificity (89.2%), and NPV (98.8%). Owing to its high sensitivity and specificity, its compatibility with minimally invasive samples (e.g., blood, urine, feces, and cervical secretions), its standardized and simple workflow, and its rapid turnaround time, DNA methylation testing holds strong potential for use in routine clinical screening and population-based early detection of endometrial cancer, significantly enhancing screening adherence and scalability. Notably, one woman with uterine carcinosarcoma had a negative methylation result, likely because an insufficient number of exfoliated cells were collected or because the disease was in the transition period, during which the patient responded well to estrogen treatment. This aspect still needs further consideration and verification.

The three methylation markers identified in this study (CDO1, CELF4, and NEFM) each have distinct biological roles that may underpin their diagnostic utility in endometrial cancer. CDO1 (cysteine dioxygenase type 1) encodes a key enzyme in cysteine catabolism and functions as a tumor suppressor. Promoter hypermethylation of CDO1 leads to transcriptional silencing, disrupting cysteine metabolism and facilitating tumor cell survival under oxidative stress. CDO1 hypermethylation has been reported across multiple cancer types and is associated with poor prognosis in several malignancies, suggesting its silencing confers a selective growth advantage during malignant transformation (41). CELF4 (CUGBP Elav-like family member 4) is an RNA-binding protein involved in post-transcriptional regulation of mRNA stability and splicing. Its methylation-associated silencing may impair the post-transcriptional control of tumor suppressor transcripts, thereby contributing to oncogenic dysregulation (42). NEFM (neurofilament medium chain) has been identified as a methylation marker in gynecological malignancies, though its precise functional role in endometrial tumorigenesis remains to be fully elucidated (43). The hypermethylation of these genes likely reflects broad epigenetic reprogramming events occurring early in endometrial carcinogenesis, consistent with the known role of promoter CpG island hypermethylation as an early and frequent event in tumor development. It should be noted that the present study was designed as a cross-sectional diagnostic accuracy study, and therefore data on the correlation of these methylation markers with tumor grade, FIGO stage, molecular subtype (e.g., POLE-ultramutated, mismatch repair-deficient, copy-number-high, or copy-number-low), or patient prognosis were not systematically collected or analyzed. Whether the methylation levels of CDO1, CELF4, and NEFM differ across these biologically distinct subgroups and whether they retain diagnostic utility in non-endometrioid or high-grade tumors remains an important open question. Similarly, the potential of these markers to detect premalignant lesions such as endometrial intraepithelial neoplasia (EIN) was not a primary endpoint of this study, given the limited number of EIN cases enrolled. These questions represent meaningful directions for future prospective studies with larger and more clinically diverse cohorts.

The Cervex-Brush is a sampling tool designed to simultaneously collect cells from the ectocervix and endocervix, at a site anatomically proximal to the uterine cavity. This proximity is likely to facilitate the capture of a higher proportion of shed endometrial cells compared with vaginal swabs, in which endometrial cell content may be more variable due to dilution by vaginal secretions and greater anatomical distance from the uterus. From a clinical applicability standpoint, cervical scraping with the Cervex-Brush can be seamlessly integrated into existing gynecological examination workflows, including routine cervical cancer screening visits without additional patient burden, making it a practical and readily implementable sampling strategy.

Despite the promising results, this study has several limitations. First, the findings may be constrained by the methodology and/or the sample collection procedures used and by the variability inherent to both pathological assessment and qPCR-based methylation detection. The latter variability includes bisulfite conversion efficiency and inter-run differences; the absolute methylation values obtained by qPCR may not be directly comparable to those obtained using alternative platforms such as Whole Genome Bisulfite Sequencing or methylation arrays. Second, the study cohort was limited to Chinese women who were treated at a single center, and subgroup analyses stratified by tumor histological grade and FIGO stage were not performed due to limited statistical power and the absence of systematic staging data as a primary endpoint. Third, whether clinical factors influence the sensitivity of the methylation marker analysis warrants investigation in future studies involving larger, prospectively annotated cohorts. Ongoing validation through the large multicenter EndoMethy-II-IV study will help determine whether the high diagnostic accuracy observed here can be replicated in diverse clinical settings.

## Conclusions

As a noninvasive method for detecting endometrial cancer, DNA methylation biomarkers (*CDO1^m^*, *CELF4^m^*, and *NEFM^m^*) in cervical cytology have demonstrated great accuracy. Incorporating the endometrial thickness of TVS could slightly improve the specificity but decrease the sensitivity. A large-scale study is needed to clarify the potential role of DNA methylation in detecting endometrial cancer as an independent strategy.

## Data Availability

The original contributions presented in the study are included in the article/**Supplementary Material**. Further inquiries can be directed to the corresponding author.
