# Supplementary material for: Development and validation of hypermethylated gene markers in cervical cytological samples for detecting endometrial cancer (EndoMethy-I trial)
Source: Front Oncol. 2026 Jun 19;16:1849730. doi: 10.3389/fonc.2026.1849730 (PMC13328268; doi:10.3389/fonc.2026.1849730)
Supplement: Supplementary file 1 [file Table1.docx]

**INDEX**

[Supplementary Table 1. Specimen characteristics for methylation chip testing. 2](#_Toc5618)

[Supplementary Table 2. Candidate CpG sites identified by Lasso Logistic Regression using the 850k Methylation Array. 3](#_Toc27296)

[Supplementary Table 3. The primer sets of the methylation genes in the training set. 4](#_Toc5298)

[Supplementary Table 4. AUC values and ΔCt thresholds for each gene identified through Lasso Logistic Regression in the training set. 5](#_Toc23702)

[Supplementary Table 5. The epidemiological and clinical characteristics of the participants in the training set. 6](#_Toc14159)

[Supplementary Table 6. The accuracy of clinical and methylation indexes for detecting endometrial cancer in the training set. 7](#_Toc16872)

[Supplementary Table 7. The epidemiological and clinical characteristics of the participants in the validation set. 8](#_Toc31744)

[Supplementary Table 8. The accuracy of clinical and methylation indexes for detecting endometrial cancer in the validation set. 9](#_Toc386)

[Supplementary Table 9. The definition of decision tree for endometrial cancer diagnosis with various screening strategies. 10](#_Toc20810)

# Supplementary Table 1. Specimen characteristics for methylation chip testing.

| **Sample types** | **Endometrial subtype2** | **Number of samples** | **Histology** |
| --- | --- | --- | --- |
| Cervical scrapings | I+II | 2 | EC (H)+CC; EC (M)+UC |
| Cervical scrapings | I+II | 3 | EC (L)+CC; EC (L)+UC |
| Cervical scrapings | II | 2 | CC; SC |
| Cervical scrapings | I | 5 | EC (H) |
| Cervical scrapings | I | 5 | EC (H) |
| Cervical scrapings | I | 2 | EC (H); EC (H-M) |
| Cervical scrapings | I | 2 | EC (H); EC (H-M) |
| Cervical scrapings | Benign | 10 | PE; SE |
| Cervical scrapings | Benign | 9 | AE; EP |
| Endometrial tissue | I+II | 2 | EC (H)+CC; EC (M)+UC |
| Endometrial tissue | I+II | 3 | EC (L)+CC; EC (L)+UC |
| Endometrial tissue | II | 2 | CC; SC |
| Endometrial tissue | I | 5 | EC (H) |
| Endometrial tissue | I | 5 | EC (H) |
| Endometrial tissue | I | 2 | EC (H); EC (H-M) |
| Endometrial tissue | I | 2 | EC (H); EC (H-M) |
| Endometrial tissue | Benign | 10 | PE; SE |
| Endometrial tissue | Benign | 9 | AE; EP |

AE, atrophic endometrium; CC, clear cell carcinoma; EC (H), highly differentiated endometrial carcinoma; EC (M), medium differentiated endometrial carcinoma; EC (L), low differentiated endometrial carcinoma; EP, endometrial polyps; PE, proliferative endometrium; SC: serous carcinoma; SE: secretory endometrium; UC: uterine carcinosarcoma.

# Supplementary Table 2. Candidate CpG sites identified by Lasso Logistic Regression using the 850k Methylation Array.

| **CpG** | **Coefficient** | **Position_annotation** | **Gene** | **Position** | **AlleleA_ProbeSeq** |
| --- | --- | --- | --- | --- | --- |
| cg01761362 | -1.27E+00 | Body | *ACLY* | chr17:40051005-40051655 | TAACTAACAAAATAAATAAATCAAAAACATATACACRTACRTCACCTAAC |
| cg12150111 | -6.30E-02 | TSS200 | *LOC100133985* | chr6:5084385-5085895 | ACCACAACTAAAAACCAAAAACTCCAAAATAAAACTACATTATCTTATCA |
| cg16265906 | 3.15E-01 | 1stExon;5'UTR | *CDO1* | chr5:115151805-115152735 | AACCCRAAAAAATAAACCCCACAAATCTAAACCTAATTTATATATACACC |
| cg19810457 | -5.58E-05 | Body;3'UTR | *MAPK9;MAPK9* | chr5:180247355-180247357 | AAAACCCCCCTTAAAACACAATCTAAAATAAATTTTACRACTTTATCCTC |
| cg08138379 | 5.68E-02 | 5'UTR | *AP1G2* | chr14:24035925-24036635 | ACCTATAAAACCCRAACTCTTAAAATCTTAACACAAAACRAACCTAAACC |
| cg17078116 | 8.92E-02 | TSS200;1stExon | *NEFM* | chr8:24771805-24773195 | ACTAAAATCAATACACAACACCAAAAAATCCCTAAAACAACAACTCAACA |
| cg25091829 | 5.23E-04 | 1stExon;5'UTR | *TSPYL1;TSPYL1* | chr6:116601020-116601295 | TAAAAATCTCACAATATCACAAAAACTTACTAAACCCAAAATTAATTCCA |
| cg23098131 | 8.03E-02 | TSS200 | *G3BP2* | chr4:76598565-76598830 | ACTCCACAAAAAAACACAAAAAAATTAAATCCAACTCTAAAAAAAATACA |
| cg12150111 | 2.41E-04 | TSS200 | *PPP1R3G* | chr6:5084385-5085895 | ACCACAACTAAAAACCAAAAACTCCAAAATAAAACTACATTATCTTATCA |
| cg14029832 | 9.26E-02 | Body | *CELF4* | chr18:35033605-35034224 | AAAAACAAATCATATCCCTTTACATCCCAACAAATAAACAATAATTTCTC |
| cg03893387 | 2.48E-03 | Body | *MX1* | chr9:129432660-129433015 | ATATATATACATACCTATATTACATATACCAACAATCTAAACCCTCACCC |

# Supplementary Table 3. The primer sets of the methylation genes in the training set.

| ACLY-F | ATGGATAGGTTGAGAGTTCGAT |
| --- | --- |
| ACLY-FP | TTCGTATAGGTAGAGTCGTTCGTTTTCGG |
| ACLY-R | AAACACCTTTCCCTCTTATATAAC |
| LOC-F | ATTTGTTAAATAATAAAAGATATT |
| LOC-FP | TCGAAGTAAAAAGATAAGGTATAGATCGA |
| LOC-R | CAATTATATACATAACAAACATCTTC |
| *CDO1*-F | GTTTATATTTTTAAGTTATCG |
| *CDO1*-FP | ATTTCGGGCGCGGAGATGCGG |
| *CDO1*-R | ACCCTCTACTAATCCG |
| MAPK9-F | GTTTGTATTTGTAGTTCGAGG |
| MAPK9-FP | TTCGCGGATTTCGCGGTGTGAG |
| MAPK9-R | GAAAACCGAACTAAAAACG |
| AP1G2-F | TATGTTTAATAGGAAGGGGCG |
| AP1G2-FP | GGTGCGGACGTATGCGTAGTG |
| AP1G2-R | ATAAATCTCCAAATTCCGC |
| *NEFM*-F | ATAAGTTTTTCGGGTGGTTG |
| *NEFM*-FP | ATTCGTCGATTTGGGCGTTG |
| *NEFM*-R | CTATAACACCGACAACACCAAA |
| TSPYL1-F | GTTGGTTGGTAGGGAGCG |
| TSPYL1-FP | AAGTTTTCGCGATATCGTG |
| TSPYL1-R | ATATCGTCAAAACTAAAAATCTC |
| G3BP2-F | GTTTTAGGGTTTATAGAGTTGC |
| G3BP2-FP | GCGCGGTCGTCGTTAGTTCGGT |
| G3BP2-R | CCAACTCAATCCCCATTAC |
| PPP1R3G-F | GGCGGGGAGGGTGTTTG |
| PPP1R3G-FP | GGCGTTGGCGTTCGGTTGCGG |
| PPP1R3G-R | CACGCACCACCTCCCA |
| *CELF4*-F | GTATATAAAGATGGTTACG |
| *CELF4*-FP | TAACGGGTTCGGTAGTAGTT |
| *CELF4*-R | AACTATAACTTAATCCG |
| MX1-F | GGAGGATGGGTAGGTTGTG |
| MX1-FP | TTCGTCGTTTCGCGATTATTCGTT |
| MX1-R | CTCCCCCAACCTAAACC |

# Supplementary Table 4. AUC values and ΔCt thresholds for each gene identified through Lasso Logistic Regression in the training set.

| **Genes** | **AUC** | **Threshold** |
| --- | --- | --- |
| *CDO1* | 0.925865 | 10.1 |
| *NEFM* | 0.9134735 | 8.5 |
| *CELF4* | 0.8941235 | 8.9 |
| ACLY | 0.8751311 | 9.3 |
| G3BP2 | 0.8390525 | 7.4 |
| LOC100133985 | 0.8371461 | 9.6 |
| MAPK9 | 0.8166524 | 8.9 |
| PPP1R3G | 0.8139834 | 6.6 |
| AP1G2 | 0.813626 | 6.8 |
| TSPYL1 | 0.7925841 | 7.2 |
| MX1 | 0.6642837 | 7.4 |

AUC, area under the curve.

# Supplementary Table 5. The epidemiological and clinical characteristics of the participants in the training set.

|  | **Non-endometrial cancer** | **Endometrial cancer** | ***p* value** |
| --- | --- | --- | --- |
| Participants (n) | 269 | 78 |  |
| Age (years, median [SD]) | 46.20 (11.35) | 51.97 (10.76) | <0.001 |
| Gravidity |  |  | 0.536 |
| 0 | 68 (25.3) | 13 (16.7) |  |
| ≥1 | 201 (74.7) | 65 (83.3) |  |
| Parity |  |  | 0.152 |
| 0 | 68 (25.3) | 13 (16.7) |  |
| ≥1 | 201 (74.7) | 65 (83.3) |  |
| BMI at diagnosis (kg/m^2^) |  |  | <0.001 |
| <25 | 189 (70.3) | 30 (38.5) |  |
| 25-30 | 68 (25.3) | 32 (41.0) |  |
| ≥30 | 12 (4.5) | 16 (20.5) |  |
| Presenting complaint |  |  | <0.001 |
| Post-menopause |  |  |  |
| PMB | 18 (6.7) | 35 (44.9) |  |
| No symptoms | 41 (15.2) | 7 (9.0) |  |
| Pre-menopause | 55 (67.9) | 19 (44.2) |  |
| Abnormal bleeding | 115 (42.8) | 29 (37.2) |  |
| No symptoms | 95 (35.3) | 7 ( 9.0) |  |
| Endometrial thickness |  |  | <0.001 |
| Post-menopause |  |  |  |
| ≥5 mm | 19 ( 7.1) | 31 (39.7) |  |
| <5 mm | 40 (14.9) | 11 (14.1) |  |
| Pre-menopause |  |  |  |
| ≥11 mm | 56 (20.8) | 12 (15.4) |  |
| <11 mm | 154 (57.2) | 24 (30.8) |  |
| Diabetes |  |  | 0.097 |
| Yes | 12 (4.5) | 8 (10.3) |  |
| No | 257 (95.5) | 70 (89.7) |  |
| PCOS |  |  | 0.383 |
| Yes | 103 (38.3) | 25 (32.1) |  |
| No | 166 (61.7) | 53 (67.9) |  |
| CA125 (U/ml) |  |  | <0.001 |
| <35 | 183 (68.0) | 52 (66.7) |  |
| ≥35 | 51 (19.0) | 26 (33.3) |  |
| Not available | 35 (13.0) | 0 (0.0) |  |

BMI, body mass index; PCOS, polycystic ovary syndrome; PMB, post-menopausal bleeding.

# Supplementary Table 6. The accuracy of clinical and methylation indexes for detecting endometrial cancer in the training set.

| **Performance** | **Sensitivity**  **% (95% CI)** | **Specificity**  **% (95% CI)** | **AUC**  **% (95% CI)** | **LR+**  **(95% CI)** | **LR-**  **(95% CI)** | **PPV**  **% (95% CI)** | **NPV**  **% (95% CI)** | **OR**  **(95% CI)** |
| --- | --- | --- | --- | --- | --- | --- | --- | --- |
| *CDO1^m^*(+) | 85.9 (76.17-92.74) | 90.71 (86.59-93.9) | 88.3 (84.04-88.3) | 9.24 (9.13-9.35) | 0.16 (0.04-0.27) | 72.83 (62.55-81.58) | 95.69 (92.41-97.83) | 59.45 (27.83-126.98) |
| *CELF4^m^*(+) | 75.64 (64.6-84.65) | 95.54 (92.34-97.67) | 85.59 (80.64-85.59) | 16.96 (16.84-17.08) | 0.25 (0.14-0.37) | 83.1 (72.34-90.95) | 93.12 (89.46-95.8) | 66.5 (30.6-144.52) |
| *NEFM^m^*(+) | 73.08 (61.84-82.5) | 95.54 (92.34-97.67) | 84.31 (79.2-84.31) | 16.38 (16.26-16.5) | 0.28 (0.16-0.4) | 82.61 (71.59-90.68) | 92.45 (88.68-95.26) | 58.13 (27.05-124.94) |
| *CDO1^m^* or *CELF4^m^*(+) | 92.31 (84.01-97.12) | 89.22 (84.89-92.66) | 90.76 (87.26-90.76) | 8.56 (8.47-8.66) | 0.09 (0-0.18) | 71.29 (61.43-79.85) | 97.56 (94.77-99.1) | 99.31 (39.67-248.62) |
| *CDO1^m^* or *NEFM^m^*(+) | 91.03 (82.38-96.32) | 90.71 (86.59-93.9) | 90.87 (87.23-90.87) | 9.79 (9.7-9.89) | 0.1 (0-0.2) | 73.96 (64-82.38) | 97.21 (94.34-98.87) | 98.99 (41.11-238.38) |
| *CELF4^m^* or *NEFM^m^*(+) | 84.62 (74.67-91.79) | 93.68 (90.07-96.28) | 89.15 (84.86-89.15) | 13.39 (13.28-13.5) | 0.16 (0.06-0.27) | 79.52 (69.24-87.59) | 95.45 (92.19-97.63) | 81.53 (37.11-179.13) |
| *CDO1^m^* or *CELF4^m^ or NEFM^m^*(+) | 96.15 (89.17-99.2) | 89.22 (84.89-92.66) | 92.69 (89.85-92.69) | 8.92 (8.84-9) | 0.04 (0-0.12) | 72.12 (62.47-80.46) | 98.77 (96.43-99.74) | 206.9 (61.29-698.45) |
| CA125 ≥35 U/ml | 53.85 (42.18-65.21) | 69.89 (64.02-75.31) | 61.87 (55.66-61.87) | 1.79 (1.62-1.95) | 0.66 (0.49-0.83) | 34.15 (25.84-43.24) | 83.93 (78.45-88.48) | 2.71 (1.62-4.54) |
| BMI ≥25 kg/m^2^ | 61.54 (49.83-72.34) | 69.52 (63.64-74.96) | 65.53 (59.44-65.53) | 2.02 (1.86-2.18) | 0.55 (0.39-0.72) | 36.92 (28.63-45.83) | 86.18 (80.86-90.47) | 3.65 (2.16-6.17) |
| BMI ≥30 kg/m^2^ | 20.51 (12.2-31.16) | 95.54 (92.34-97.67) | 58.03 (53.35-58.03) | 4.6 (4.48-4.71) | 0.83 (0.72-0.95) | 57.14 (37.18-75.54) | 80.56 (75.79-84.76) | 5.53 (2.49-12.28) |
| Abnormal endometrial thickness | 55.13 (43.44-66.41) | 72.12 (66.35-77.39) | 63.62 (57.45-63.62) | 1.98 (1.81-2.14) | 0.62 (0.46-0.79) | 36.44 (27.78-45.8) | 84.72 (79.39-89.12) | 3.18 (1.89-5.34) |
| Premenopausal abnormal endometrial thickness | 15.38 (8.21-25.33) | 79.18 (73.83-83.87) | 47.28 (42.58-47.28) | 0.74 (0.61-0.87) | 1.07 (0.94-1.2) | 17.65 (9.47-28.8) | 76.34 (70.91-81.21) | 0.69 (0.35-1.37) |
| Postmenopausal abnormal endometrial thickness | 39.74 (28.83-51.46) | 92.94 (89.19-95.69) | 66.34 (60.66-66.34) | 5.63 (5.49-5.77) | 0.65 (0.51-0.79) | 62 (47.17-75.35) | 84.18 (79.52-88.13) | 8.68 (4.53-16.63) |

The positive results of methylated *CDO1, CELF4*, and *NEFM* is defined as ΔCp ≤8.4, ≤8.8 and ΔCp ≤ 8.8. Pre- and postmenopausal abnormal endometrial thickness were defined as ≥ 1 mm and ≥5 mm, respectively. AUC, area under curve; BMI, body mass index; CA125, cancer antigen 125; LR, likelihood ratio; NPV, negative predictive value; OR, odds ratio; PPV, positive predictive value.

#

# Supplementary Table 7. The epidemiological and clinical characteristics of the participants in the validation set.

|  | **Non-endometrial cancer** | **Endometrial cancer** | ***p* value** |
| --- | --- | --- | --- |
| Participants (n) | 128 | 21 |  |
| Age (years, median [SD]) | 45.80 (10.61) | 53.85 (10.96) | 0.002 |
| Gravidity |  |  | 1 |
| 0 | 20 (15.6) | 3 (14.3) |  |
| ≥1 | 108 (84.4) | 18 (85.7) |  |
| Parity |  |  | 0.81 |
| 0 | 31 (24.2) | 4 (19.0) |  |
| ≥1 | 97 (75.8) | 17 (81.0) |  |
| BMI at diagnosis (kg/m^2^) |  |  | 0.025 |
| <25 | 90 (70.3) | 10 (47.6) |  |
| 25-30 | 32 (25.0) | 7 (33.3) |  |
| ≥30 | 6 ( 4.7) | 4 (19.0) |  |
| Presenting complaint |  |  | <0.001 |
| Post-menopause |  |  |  |
| PMB | 8 ( 6.2) | 13 (61.9) |  |
| No symptoms | 21 (16.4) | 1 ( 4.8) |  |
| Pre-menopause | 55 (67.9) | 19 (44.2) |  |
| Abnormal bleeding | 59 (46.1) | 5 (23.8) |  |
| No symptoms | 40 (31.2) | 2 ( 9.5) |  |
| Endometrial thickness |  |  | <0.001 |
| Post-menopause |  |  |  |
| ≥5 mm | 11 ( 8.6) | 12 (57.1) |  |
| <5 mm | 18 (14.1) | 2 ( 9.5) |  |
| Pre-menopause |  |  |  |
| ≥11 mm | 28 (21.9) | 5 (23.8) |  |
| <11 mm | 71 (55.5) | 2 ( 9.5) |  |
| Diabetes |  |  | 0.013 |
| Yes | 4 ( 3.1) | 4 (19.0) |  |
| No | 124 (96.9) | 17 (81.0) |  |
| PCOS |  |  | 0.754 |
| Yes | 57 (44.5) | 8 (38.1) |  |
| No | 71 (55.5) | 13 (61.9) |  |
| CA125 (U/ml) |  |  | 0.014 |
| <35 | 89 (80.2) | 11 (52.4) |  |
| ≥35 | 22 (19.8) | 10 (47.6) |  |
| Not available | 17 (13.3) | 0 (0.0) |  |

BMI, body mass index; PCOS, polycystic ovary syndrome; PMB, post-menopausal bleeding.

# Supplementary Table 8. The accuracy of clinical and methylation indexes for detecting endometrial cancer in the validation set.

| **Performance** | **Sensitivity**  **% (95% CI)** | **Specificity**  **% (95% CI)** | **AUC**  **% (95% CI)** | **LR+**  **(95% CI)** | **LR-**  **(95% CI)** | **PPV**  **% (95% CI)** | **NPV**  **% (95% CI)** | **OR**  **(95% CI)** |
| --- | --- | --- | --- | --- | --- | --- | --- | --- |
| *CDO1^m^*(+) | 71.43 (47.82-88.72) | 88.28 (81.41-93.29) | 79.85 (69.57-79.85) | 6.1 (5.85-6.34) | 0.32 (0.07-0.57) | 50 (31.3-68.7) | 94.96 (89.35-98.13) | 18.83 (6.34-55.97) |
| *CELF4^m^*(+) | 66.67 (43.03-85.41) | 92.97 (87.07-96.73) | 79.82 (69.25-79.82) | 9.48 (9.24-9.73) | 0.36 (0.11-0.6) | 60.87 (38.54-80.29) | 94.44 (88.89-97.74) | 26.44 (8.52-82.06) |
| *NEFM^m^*(+) | 76.19 (52.83-91.78) | 96.09 (91.12-98.72) | 86.14 (76.66-86.14) | 19.5 (19.29-19.72) | 0.25 (0.03-0.46) | 76.19 (52.83-91.78) | 96.09 (91.12-98.72) | 78.72 (20.52-302.02) |
| *CDO1^m^* or *CELF4^m^*(+) | 76.19 (52.83-91.78) | 86.72 (79.59-92.07) | 81.45 (71.67-81.45) | 5.74 (5.5-5.98) | 0.27 (0.03-0.52) | 48.48 (30.8-66.46) | 95.69 (90.23-98.59) | 20.89 (6.77-64.46) |
| *CDO1^m^* or *NEFM^m^*(+) | 85.71 (63.66-96.95) | 87.5 (80.5-92.68) | 86.61 (78.42-86.61) | 6.86 (6.65-7.06) | 0.16 (0-0.37) | 52.94 (35.13-70.22) | 97.39 (92.57-99.46) | 42 (11.11-158.77) |
| *CELF4^m^* or *NEFM^m^*(+) | 80.95 (58.09-94.55) | 91.41 (85.14-95.63) | 86.18 (77.24-86.18) | 9.42 (9.2-9.64) | 0.21 (0-0.42) | 60.71 (40.58-78.5) | 96.69 (91.75-99.09) | 45.2 (12.92-158.15) |
| *CDO1^m^* or *CELF4^m^ or NEFM^m^*(+) | 85.71 (63.66-96.95) | 85.94 (78.69-91.45) | 85.83 (77.58-85.83) | 6.1 (5.89-6.31) | 0.17 (0-0.38) | 50 (32.92-67.08) | 97.35 (92.44-99.45) | 36.67 (9.8-137.25) |
| CA125 ≥35 U/ml | 42.86 (21.82-65.98) | 64.06 (55.11-72.35) | 53.46 (41.84-53.46) | 1.19 (0.9-1.49) | 0.89 (0.6-1.19) | 16.36 (7.77-28.8) | 87.23 (78.76-93.23) | 1.34 (0.52-3.41) |
| BMI ≥25 kg/m^2^ | 52.38 (29.78-74.29) | 69.53 (60.78-77.35) | 60.96 (49.3-60.96) | 1.72 (1.43-2.01) | 0.68 (0.39-0.98) | 22 (11.53-35.96) | 89.9 (82.21-95.05) | 2.51 (0.99-6.4) |
| BMI ≥30 kg/m^2^ | 23.81 (8.22-47.17) | 95.31 (90.08-98.26) | 59.56 (50.05-59.56) | 5.08 (4.86-5.3) | 0.8 (0.58-1.02) | 45.45 (16.75-76.62) | 88.41 (81.86-93.23) | 6.35 (1.74-23.23) |
| Abnormal endometrial thickness | 80.95 (58.09-94.55) | 69.53 (60.78-77.35) | 75.24 (65.75-75.24) | 2.66 (2.41-2.9) | 0.27 (0.03-0.52) | 30.36 (18.78-44.1) | 95.7 (89.35-98.82) | 9.7 (3.06-30.7) |
| Premenopausal abnormal endometrial thickness | 23.81 (8.22-47.17) | 78.12 (69.96-84.95) | 50.97 (40.97-50.97) | 1.09 (0.83-1.34) | 0.98 (0.72-1.23) | 15.15 (5.11-31.9) | 86.21 (78.57-91.91) | 1.12 (0.38-3.31) |
| Postmenopausal abnormal endometrial thickness | 57.14 (34.02-78.18) | 91.41 (85.14-95.63) | 74.27 (63.16-74.27) | 6.65 (6.39-6.91) | 0.47 (0.21-0.73) | 52.17 (30.59-73.18) | 92.86 (86.87-96.68) | 14.18 (4.9-41.04) |

The positive results of methylated *CDO1, CELF4*, and *NEFM* is defined as ΔCp ≤8.4, ≤8.8 and ΔCp ≤ 8.8. Pre- and postmenopausal abnormal endometrial thickness were defined as ≥1 mm and ≥5 mm, respectively. AUC, area under curve; BMI, body mass index; CA125, cancer antigen 125; LR, likelihood ratio; NPV, negative predictive value; OR, odds ratio; PPV, positive predictive value.

# Supplementary Table 9. The definition of decision tree for endometrial cancer diagnosis with various screening strategies.

| **Program** | **Hysteroscopy (%)** | **Missed cancer cases** | **Reduced missing cases (%)** |
| --- | --- | --- | --- |
| Current clinical practice | 282 (56.8) | 48 | 0 |
| Strategy 1 | 282 (56.8) | 20 | 58.3 |
| Strategy 2 | 238 (48.0) | 1 | 97.9 |
| Strategy 3 | 232 (46.8) | 2 | 95.8 |
| Strategy 4 | 230 (46.4) | 3 | 93.8 |
| Strategy 5 | 124 (25.0) | 5 | 89.6 |
| Strategy 6 | 113 (22.8) | 12 | 73.9 |
| Strategy 7 | 111 (22.4) | 12 | 73.9 |

Current clinical practice: Women were treated according to bleeding symptoms and endometrial thickness of TVS.

Strategy 1: Women with AUB accepted methylation assessment of *CDO1*, *CELF4* and *NEFM*.

Strategy 2: All women accepted methylation assessment of *CDO1*, *CELF4*, and *NEFM*, and TVS.

Strategy 3: All women accepted methylation assessment of *CDO1* and *CELF4*, and TVS.

Strategy 4: All women accepted methylation assessment of *CDO1* and *NEFM*, and TVS.

Strategy 5: All women accepted methylation assessment of *CDO1*, *CELF4* and *NEFM*.

Strategy 6: All women accepted methylation assessment of *CDO1* and *CELF4*.

Strategy 7: All women accepted methylation assessment of *CDO1* and *NEFM*.

AUB, abnormal uterine bleeding; TVS, transvaginal ultrasound.
